# Quantification of torque teno virus (TTV) DNA in saliva and plasma samples in patients at short time before and after kidney transplantation

**DOI:** 10.1080/20002297.2021.2008140

**Published:** 2021-12-12

**Authors:** Alexandre Mendes Batista, Matheus W. Caetano, Maria A. Stincarelli, Ana C. Mamana, Rodrigo Melim Zerbinati, Dmitry J. S. Sarmento, Marina Gallottini, Rafael A. V. Caixeta, José Medina-Pestana, Bengt Hasséus, Louise Zanella, Tania R. Tozetto-Mendoza, Simone Giannecchini, Paulo H. Braz-Silva

**Affiliations:** a Laboratory of Virology, Institute of Tropical Medicine of São Paulo, University of São Paulo School of Medicine, São Paulo, Brazil; b Department of Stomatology, University of São Paulo School of Dentistry, São Paulo, Brazil; c Department of Experimental and Clinical Medicine, University of Florence, Florence, Italy; d Department of Oral Medicine, State University of Paraiba, Araruna, Brazil; e Division of Renal Transplantation, Kidney and Hypertension Hospital, Federal University of São Paulo School of Medicine, São Paulo, Brazil; f Department of Oral Medicine and Pathology, University of Gothenburg Institute of Odontology, Gothenburg, Sweden; g Laboratory of Integrative Biology (LIBi), Scientific and Technological Bioresource Nucleus – Center for Excellence in Translational Medicine (BIOREN - CEMT), Universidad de La Frontera, Temuco, Chile

**Keywords:** Saliva, immunosuppression, solid organ transplantation, torque teno virus

## Abstract

**Background:**

Several reports have proposed that the viral load of torque teno virus (TTV) in plasma is a biomarker of immune function in solid organ transplantation (SOT) and in allogeneic hematopoietic stem cell transplantation. Additionally, for the latter one, TTV-DNA quantification in saliva has also been suggested.

**Aim:**

to investigate the correlation between the TTV viral load and immune function in paired saliva and plasma samples in patients on kidney transplantation.

**Materials and Methods:**

TTV-DNA viral load was quantified in paired samples of saliva and plasma from 71 patients before and a short-time after renal-transplantation by real-time PCR.

**Results:**

The data obtained from 213 paired samples showed a slight consistency in the comparison between saliva and plasma, with prevalence of TTV-DNA being 58%, 52% and 60% in saliva samples and 60%, 73% and 90% in plasma samples before and at 15–20 and 45–60 days after transplantation, respectively. Additionally, a high TTV viral load was observed in plasma at 15–20 and 45–60 days after transplantation compared to that observed in saliva at the same time.

**Conclusions:**

Overall, monitoring TTV-DNA in saliva samples could be an additional fast non-invasive option to assess the immune functionality in SOT populations.

## Introduction

Solid organ transplantation (SOT) is the best treatment option in patients with end-stage renal failure, presenting several advantages in comparison with dialysis [1]. SOT recipients are submitted to different immunosuppressive therapy regimens to avoid organ rejection, which increases the risk of infections [2]. Thus, the paucity of consistent markers for evaluating the status of immune function in SOT recipients remains a main concern [2]. The level of immunosuppressive treatment has to be balanced between rejection and opportunistic infections, which may occur in solid organ transplantation recipients [2]. Increasing evidence has suggested that measuring the torque teno virus (TTV) load after immunosuppressive treatment may be a useful tool to evaluate the efficacy of immunosuppression [3,4].

TTV is a naked, small virus with circular single-strand DNA genome, discovered in 1997 [5,6], comprising to date at least 29 genetically different species included in the genus *Alphatorquevirus* within the *Anelloviridae* family [7–9]. TTV possesses several characteristics, such as its presence as a main virus of the human virome, high viral load in immunosuppressed patients compared to healthy ones, considerable genetic diversity and lack of association with any human illness [8–11].

Several studies have investigated the viral load and kinetics of TTV in human plasma by associating the former with immune status, presence of infection and/or organ rejection after transplantation [12–22]. De Vlaminck et al. have for the first time presented evidence of a relationship between human virome, immune functionality and specific immunomodulatory treatment with potential implication for the prediction of immunocompetence in SOT recipients [22].

To date, although T cells are considered to be the main site of TTV replication [23,24], other cell types may allow viral replication [21]. Additionally, among the body fluids examined, some studies have reported that TTV DNA is highly present in the saliva [25–28]. Therefore, saliva has gained an increased interest as a non-invasive screening approach to assess the TTV DNA viral load [28]. TTV DNA has been recently investigated in the saliva and compared with paired plasma samples obtained during allogeneic hematopoietic stem cell transplantation [29]. In order to provide clinical information in SOT recipients, this study aimed to investigate the TTV viral load in saliva and paired plasma samples in patients treated with renal transplantation.

## Patients and methods

### Patients and samples

This is a cohort study in which patients attending a renal transplant center (Renal Transplant Unit of the Federal University of São Paulo Kidney and Hypertension Hospital, Brazil) were included, all older than 18 and submitted to single kidney transplantation. Exclusion criteria were described in a previously published study by our group as follows: being submitted to dual kidney or multi-organ transplantation, undergoing immunosuppressive therapy prior to inclusion, being HIV-seropositive or presenting cognitive impairment affecting the ability to understand the informed consent form. Moreover, patients dropping out or having inconclusive blood or saliva samples for viral detection were also excluded from the study [30].

Clinical and demographic characteristics of the patients were collected during an interview. The samples (saliva and blood) were collected in three different experimental periods as follows: the first within 24 hours before renal transplantation, the second between 15 and 20 days and the third between 45 and 60 days after the surgery. Mouthwash samples were also collected from the patients, who were comfortably seated in a bright and well-ventilated room. They were instructed to make mouthwash with 5 mL of Listerine® solution for 30 seconds and then to put the fluid into a 50-ml Falcon tube. Blood samples were collected by nurses at the same moment of the saliva collection. After collection and identification, the samples were centrifuged at 800 rpm in a conical tube and then 200 μL of each sample were aliquoted into cryotubes for storage in a freezer at −80°C.

This study was approved by the Research Ethics Committee of the University of São Paulo School of Dentistry according to protocol number 1,824,857 and followed the ethical standards set by the Declaration of Helsinki. All the participants signed an informed consent form.

### Quantification of TTV DNA

DNA extraction was performed in saliva and plasma samples by using a genomic DNA extraction kit (Real Genomics ™, Real Biotech Corporation) according to the manufacturer’s instructions. All samples were found to be suitable for DNA amplification. A quantitative (real-time) polymerase chain reaction (qPCR) with TTV-specific primers and a probe was performed by using a synthetic standard curve with known amounts of synthetic oligonucleotide for TTV quantification (Forward primer 5′-GTGCCGIAGGTGAGTTTA-3′; Reverse primer 5′-AGCCCGGCCAGTCC-3′; Probe: FAM5′-TCAAGGGGCAATTCGGGCT-3′MGBNFQ; Synthetic curve: 5 ′-TTCGTAGCCCGGCCAGTCCCGTATAGCCCGAATTGCCCCTTGAATGCGTTAAACTCACCAICGGCACCTGATA-3′). The resulting data were analysed by using the QuantStudio Design & Analysis Software, version 1.4.1 [31,32].

### Statistical analysis

The results were analysed with the SPSS software, version 17.0 (SPSS, Inc., Chicago, IL). The viral load showed a skewed distribution (Kolmogorov-Smirnov test, *P* < 0.001) and for this reason data were analysed by non-parametric tests. The Kruskall-Wallis test was used to observe whether viral loads in saliva and plasma were different between the immunosuppressive schemes used. Mann-Whitney’s test was used to compare differences in the viral load of TTV between patients hospitalised after kidney transplantation and outpatients. The Friedman’s test was used to compare whether the DNA copy number of TTV in saliva and plasma differed between the experimental times. Correlation between the viral load in saliva and that in plasma was assessed by Spearman’s correlation test, whereas the kappa agreement test was used to analyse the concordance between positivity for TTV in saliva and that in blood. All statistical analyses were performed at a significance level of 5%.

## Results

### Demographic and clinical characteristics of the renal transplant patients

Clinical and demographic characteristics of 71 patients admitted to the Renal Transplant Unit of the Federal University of São Paulo Kidney and Hypertension Hospital and enrolled in this study are reported in Table 1. Saliva and plasma samples were collected at three experimental times: 24 hours before transplantation, 15–20 days after transplantation and 45–60 days after transplantation. In total, 213 saliva and plasma samples were collected.

**Table 1.** Demographic and clinical characteristics of the study patients

Variable		Number of patients	%
Gender	Male (mean ± SD age: 44.1 ± 13.3 years)	36	50.7
	Female (mean ± SD age: 42.6 ± 10.6 years)	35	49.3
Skin colour	Brown	27	38
	White	24	33.8
	Black	20	28.2
Smoking	Present	2	2.8
	Never	49	69
	Past	20	28.2
Use of alcohol	Present	2	2.8
	Never	28	39.4
	Past	41	57.8
Dialysis	Haemodialysis	64	90.2
	Peritoneal	3	4.2
	Not performed	4	5.6
Donor	Living	39	54.9
	Deceased	32	45.1
Relationship ^a^	Relatives None	39 42	55 45
Underlying disease of renal failure	Hypertension	20	28.2
	Diabetes mellitus	11	15.5
	Glomerulonephritis	10	14.1
	Polycystic kidneys	5	7.0
	IgA nephropathy	3	4.2
	Systemic lupus	2	2.8
	Alport syndrome	2	2.8
	Others	7	9.9
	Undetermined	11	15.5
Immunosuppressive regimen ^b^	Tacrolimus + Sodium mycophenolate + Prednisone	31	43.7
	Tacrolimus + Azathioprine + Prednisone	21	29.6
	Tacrolimus + Everolimus + Prednisone	13	18.3
	Cyclosporine + Azathioprine + Prednisone	6	8.5
CMV IgG recipient		65	91
CMV IgG donor		50	70

^a^Relatives are: brother/sister, mother/father, husband/wife, son/daughter, husband, grandfather, cousin. ^b^ The treatment doses (mg dose/day) were: Tacrolimus 2–20, Cyclosporine 250–500, Myfortic 720–1440, Azhatioprine 75–150, Prednisone 5–40, Everolimus 2.5–6. All patients received Bactrim at a dose of 400/800 (mg dose/day).

The profile of the patients was the following (Table 1): slight majority of males (36/71; 50.7%) and mean age of 43.34 ± 11.98 years old (44.1 ± 13.3 and 42.6 ± 10.6 years for males and females, respectively). Twenty patients (28.2%) reported that they were smokers before the kidney transplantation and 57.8% had a history of alcoholism. The mean dialysis time was 39.64 ± 38.02 months, in which haemodialysis (90.2%) was the main treatment performed. Most transplant organs came from living donors (54.9%) and hypertension was the main underlying disease (28.2%) causing renal failure. The most commonly used immunosuppressive regimen was a combination of tacrolimus, sodium mycophenolate and prednisone (43.7%) (Table 1). Moreover, 65 out of 71 (90%) recipients and 50 out of 71 (71%) donors were cytomegalovirus seropositive.

### TTV DNA status in saliva and plasma samples

TTV DNA status in the paired saliva and plasma samples collected from the 71 patients before and after renal transplantation is shown in Table 2. Kappa concordance test showed that there was a minimum reliability in the comparison between saliva and plasma for detection of TTV (k = 0.336, *P* < 0.001; concordance = 69.9%). It was observed that the concordance was 52.1% for detection of TTV, whereas both saliva and plasma had a negative concordance of 17.8% (Table 2).

**Table 2.** Concordance in the detection of TTV DNA in saliva and plasma.

	Plasma					
		Positive n(%)	Negative n(%)	TOTAL	Kappa	
					k^1^	p
Saliva	Positive	111 (52.1)	16 (7.5)	127 (59.6)	0.336	<0.001*
	Negative	48 (22.5)	38 (17.8)	86 (40.4)		
	TOTAL	159 (74.6)	54 (25.4)	213 (100)		

^1^Kappa value; * Statistical significance (*P* < 0.05).

The prevalence of TTV DNA before and at 15–20 and 45–60 days after transplantation in plasma samples were 60%, 73% and 90%, whereas those in saliva samples were 58%, 52% and 60%, respectively. A significant difference between plasma and saliva viral status was confirmed at both experimental times after transplantation (*P* < 0.01, chi-square test).

**Figure 1.**
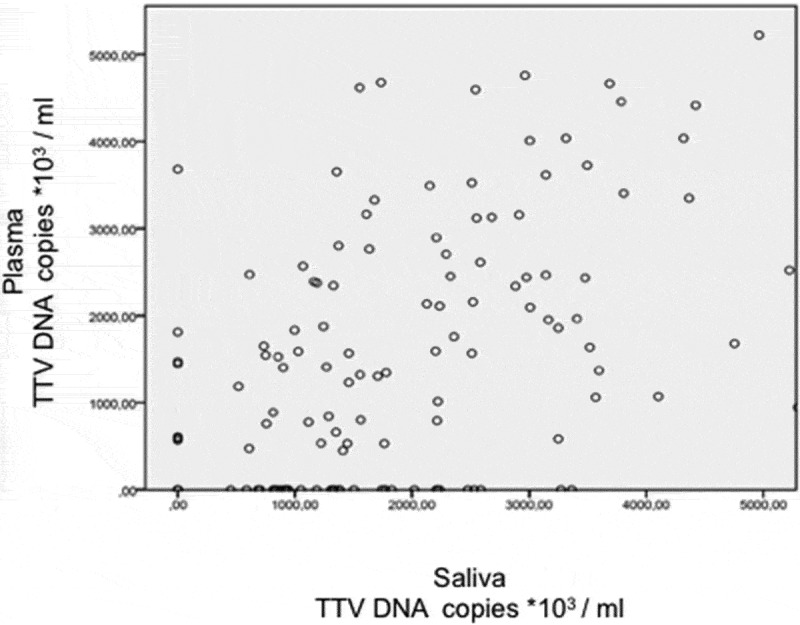
Correlation between the TTV DNA load in saliva and that in plasma. Spearman’s correlation test: N = 213; coefficient correlation = 0.323; P < 0.001)

In all TTV positive patients, the viral load showed a skewed distribution (Kolmogorov-Smirnov test, *P* < 0.001). Additionally, Spearman’s correlation test (coefficient = 0.323; *P* < 0.001) showed a weak positive correlation between the viral load of TTV found in saliva and plasma (Figure 1).

We sought to assess whether the type of immunosuppressive regimen influenced the TTV DNA status. A higher viral load in plasma was observed for a drug regimen consisting of tacrolimus, sodium mycophenolate and prednisone compared to other regimens (Table 3). The relationship between the viral load of TTV (in saliva and plasma) and hospitalisation was also analysed, showing no statistically significant difference (Mann-Whitney test, *P* > 0.05).

**Table 3.** Association of TTV viral load in saliva and plasma with immunosuppressive regimen used in the phase of maximum immunosuppression (i.e. third sample collection)

Immunosuppressive regimen	N	Rank saliva^(1)^	Rank plasma^(1)^
Tacrolimus + Sodium mycophenolate + Prednisone	31	35.45	46.42
Tacrolimus + Azathioprine + Prednisone	21	37.48	30.21
Tacrolimus + Everolimus + Prednisone	13	33.08	24.04
Cyclosporine + Azathioprine + Prednisone	6	40	28.33
*P value*		0.889	0.002*

^(1)^Kruskal-Wallis test; *Statistical significance (*P* < 0.05).

### Comparison of the TTV DNA status in saliva and plasma samples

**Figure 2.**
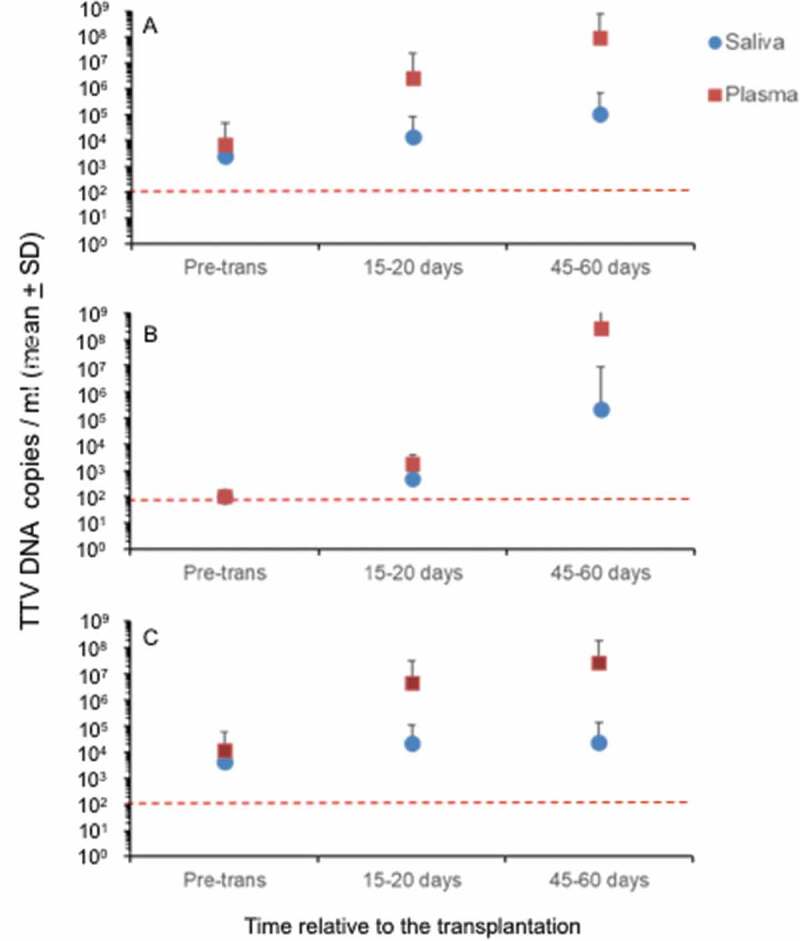
Comparison between the TTV DNA loads in plasma and saliva at the three experimental times. Viral loads in saliva and plasma were obtained by using total samples examined (a), viral load in saliva and plasma obtained by using only the samples that were negative before transplantation time (b) and viral load in saliva and plasma obtained by using only the samples that were positive before transplantation time (c) is reported. The red dashed line indicates low limit of detection of TTV DNA. The values are shown as means ± standard deviations

The comparison of TTV DNA status at different experimental times before and after transplantation in paired saliva and plasma samples is shown in Table 4 and Figure 2. Collectively, at the three different times the TTV DNA viral load in saliva samples showed a mean copies number from 2 × 10^3^ to 1 × 10^5^ copies/ml (the value ranged from 4.3 × 10^2^ to 4.3 × 10^6^ copies/ml) whereas the TTV DNA viral load in plasma samples exhibited a mean of copies number from 3 × 10^5^ to 1 × 10^9^ copies/ml (values ranged from 5.5 × 10^2^ to 5.7 × 10^9^ copies/ml). Friedman’s test showed that the DNA copy number of TTV in saliva and plasma differed between the experimental times (*P* < 0.001; Table 4). Multiple comparative tests were also performed and showed that the DNA copy number of TTV in saliva differed significantly between the second and third collections, whereas in plasma it differed statistically in the third collection compared to the first and second one (Table 4).

**Table 4.** Relationship of TTV viral load in saliva and plasma with experimental times

TTV		Viral load (copies of DNA/mL)		
		Mean ± standard deviation	Rank mean	*p*^(b)^
Saliva	Pre-transplantation	2562.58 ± 5499.85	2.04	**<** 0.001*
	15–20 days after transplantation	13,334.08 ± 69,032.64	1.64	
	45–60 days after transplantation	108,480.20 ± 557,334.40	2.32	
Plasma	Pre-transplantation	7064.19 ± 39,630.31	1.63	**<** 0.001*
	15–20 days after transplantation	2,523,052 ± 20,043,054.55	1.97	
	45–60 days after transplantation	97,210,729.23 ± 696,410,531.00	2.40	

^(a)^Indicates the number of samples studied; ^(b)^ Friedman’s test; * Statistical significance (*P* < 0.05).

Additionally, it was of interest to examine the difference of the TTV DNA load in saliva and plasma coupled samples before and after (15–20 and 45–60 days) renal transplantation. Figure 2 (panel A) shows the high increase in the TTV DNA load (mean of 2.5 × 10^6^ and 9.7 × 10^7^ copies /ml at 15–20 and 45–60 days compared to a mean of 7 × 10^3^ copies /ml at the pre-transplantation time) in plasma samples at 15–20 and 45–60 days after transplantation compared to the TTV DNA load (mean of 1.3 × 10^4^ and 1 × 10^5^ copies /ml at 15–20 and 45–60 days compared to a mean of 2.5 × 10^3^ copies /ml at the pre- transplantation time) in saliva samples at the same times. Then, the TTV DNA viral load was scheduled subdividing the total samples in the group of samples obtained from patients with a negative viral load (Figure 2(b)) and in the group of samples obtained from patients with a positive viral load before transplantation (Figure 2(c)). In this context, in presence of similar TTV viral load status before transplantation, it was observed that high differences of TTV DNA load in plasma samples (mean of 4.6 × 10^6^ copies/ml) compared to saliva samples (mean of 2.2 × 10^4^ copies/ml) was evident starting from early times (15–20 days after transplantation) in the group of samples with a positive viral load before transplantation (Figure 2(c)). Conversely, in the group of samples TTV DNA negative before transplantation, a similar TTV DNA viral load was observed in plasma samples (mean of 1.7 × 10^3^ copies/ml) and saliva samples (mean of 4.9 × 10^2^ copies/ml) at early time post-transplanted exhibiting difference only at 45–60 days post transplantation (mean of 2.6 × 10^8^ copies/ml versus 2.2 × 10^6^ copies/ml, for plasma and saliva samples, respectively) (Figure 2(b)).

## Discussion

In this study, the TTV DNA status in paired saliva and plasma samples was investigated in 71 patients before and after (15–20 and 45–60 days) renal transplantation. Data showed a statistical consistency in the comparison between saliva and plasma for detection of TTV DNA positivity (k = 0.336, *P* < 0.001, concordance = 69.9%). However, a significant difference between plasma and saliva regarding the viral status was confirmed in both experimental times after transplantation (*P* < 0.01, chi-square test). In saliva, particularly, the DNA copy number of TTV differed significantly between the second and third collections, whereas in plasma it differed significantly in the third collection compared to the first and second ones. Additionally, plasma samples at 15–20 and 45–60 days after transplantation showed high increase of the TTV DNA viral load compared to the saliva samples at the same experimental times. In this context, the TTV viral load showed no normal distribution and showed high values in the plasma of patients on a drug regimen consisting of tacrolimus, sodium mycophenolate and prednisone compared to other regimens.

Several studies reported that monitoring of the TTV DNA load in blood may predict the risk of opportunistic infections and allograft rejection events in the SOT setting [22]. In particular, it has been shown that in SOT recipients (e.g. liver, kidney and lung transplantations) the TTV viral load progressively increases during the course of the transplantation, peaking within three months post-transplantation and then declining during the following six months [16–20,33–36]. Additionally, studies have investigated the kinetics of plasma TTV DNA after allogeneic hematopoietic stem cell transplantation, thus clearly demonstrating that the TTV DNA load decreases after conditioning therapy with an increased viral load and correlating the degree of T-cell immune reconstitution following engraftment [37–39]. Noteworthy, these data support the assumption that T-cells are the major site of TTV replication [23,37–41].

To date, there are studies on TTV DNA in saliva proposing that the oral cavity is another site for TTV viral replication or transmission route, thus potentially contributing to the total viral load [24–29]. Thus, recently the TTV DNA load in saliva after allogeneic hematopoietic stem cell transplantation was used to assess the potential utility of a non-invasive and rapid biological fluid collection (thus replacing or complementing plasma sampling) for predicting lymphocyte reconstitution after engraftment [29]. In the latter study, the TTV DNA loads were significantly higher in saliva than in plasma samples, which showed a direct correlation between TTV DNA loads in saliva and plasma and lymphocyte reconstitution after engraftment [29]. In our study, the TTV viral load in saliva was constantly found to be lower than in paired plasma samples, but with a similar increasing kinetics compared to that observed in plasma after transplantation. Also, a high increase of the TTV DNA viral load was observed in plasma samples at 15–20 and 45–60 days after transplantation compared to that obtained in paired saliva samples, especially in samples with positive viral load before the transplantation. These observed differences between our study and previous ones could be related to the different clinical status of patients undergoing renal transplantation or allogeneic hematopoietic stem cell transplantation and to the drug regime used. Also, since the TTV viral load in blood has been shown to have a wide variation, it should not be excluded that the methodology used in our study for DNA extractions from saliva and quantification of TTV DNA, could have contributed to the different results reported by other studies [25,26,29]. However, our study, performed in SOT patients in the absence of haematological disorders, confirmed that the TTV viral load is mainly associated to the viral replication in the lymphocytes replication-competent cells [23,37,38]. Also, this study reported that TTV viral shedding at early time after transplantation, in the saliva of patients with negative TTV DNA before transplantation, showed a viral load similar to that observed in paired plasma. Thus, taking into account the slow increase of the viral load observed in saliva compared to that observed in plasma samples, the TTV DNA load greater than 10^5^ copies/ml in saliva could be suggestive of an immunosuppression status. Moreover, this finding pointed out additional evidence that the oral cavity at an early time point of TTV infection/reactivation is another potential site of viral replication and the main route of transmission, as described previously [24–29].

Our study is hampered by several limitations, such as low number of samples analysed during the post-transplantation period, short-time period of examination after transplantation and lack of lymphocyte count assessment for evaluation of immune functionality. Nevertheless, our data confirm that an easy and non-invasive sequential monitoring of the TTV DNA load in saliva samples can allow the assessment of the degree of immune functionality after transplant engraftment in combination with the monitoring of the plasma TTV DNA load in SOT patients. Therefore, further studies involving larger cohorts during a long time after transplantation should be conducted to confirm the potential of saliva sampling as an additional source to evaluate the TTV viral load in combination with plasma samples in SOT patients.
